# Multi-tissue transcriptomic analysis reveals that L-methionine supplementation maintains the physiological homeostasis of broiler chickens than D-methionine under acute heat stress

**DOI:** 10.1371/journal.pone.0246063

**Published:** 2021-01-27

**Authors:** Mingyung Lee, Hyesun Park, Jung Min Heo, Ho Jun Choi, Seongwon Seo

**Affiliations:** 1 Division of Animal and Dairy Sciences, Chungnam National University, Daejeon, Korea; 2 CJ Cheiljedang Co., Ltd, Seoul, Korea; West Virginia University, UNITED STATES

## Abstract

The objective of this study was to compare the effects of supplementation with two methionine isoforms, L-methionine (L-Met) or D-methionine (D-Met), on transcriptome expression in broiler chickens under acute heat stress. A total of 240 one-day-old chicks were randomly assigned to one of four treatments in a 2 × 2 factorial arrangement: thermo-neutral vs. acute heat-stress and L-Met vs. D-Met supplementation. On day 14, the heat-stressed group was exposed to 32°C for 5 h, while the others remained at 25°C. Six chicks were randomly selected per treatment and total RNA was isolated from whole blood, ileum, and liver tissues. Two RNA samples from each tissue of each treatment group were randomly selected and pooled in equal amounts. A total of 1.87 billion raw reads obtained from 36 samples (four treatments × three tissues × three composited replicates) were mapped to the reference genome build (Gallus_gallus-5.0) and used to identify differentially expressed genes (DEGs) using DESeq2. Functional enrichment of DEGs was tested using DAVID. Comparing the two isoforms of supplemented methionine, two, three, and ten genes were differentially expressed (> 1 or < -1 log_2_ fold change) in whole blood, ileum, and liver, respectively. A total of 38, 71, and 16 genes were differentially expressed in response to the interaction between heat stress and Met isoforms in the blood, ileum, and liver, respectively. Three-tissue-specific DEGs were functionally enriched for regulation of cholesterol homeostasis and metabolism, glucose metabolism, and vascular patterning. Chicks fed with L-Met had lower immune (e.g., *IL4I1* and *SERPINI1*) and intestinal angiogenic responses (e.g., *FLT1* and *FGD5*), and stable glucose and lipid metabolism (e.g., *PCK1* and *LDLR*) under heat stress conditions. In conclusion, unlike D-Met, L-Met supplementation seems to help maintain physiological homeostasis and enhances cellular defense systems against external stresses like high environmental temperature.

## Introduction

Methionine is an essential amino acid provided in animal diets since it cannot be synthesized *de novo*. Methionine plays an important role in numerous physiological processes (e.g., one-carbon metabolism and synthesis of cysteine, glutathione [GSH], taurine, and inorganic sulfur compounds) [1]. It is normally the first or second limiting amino acid; thus, an adequate supply of methionine is important in farm animal diets [2]. Methionine deficiency can reduce protein synthesis and inhibit growth.

There are two isoforms of methionine: L-methionine (L-Met) and D-methionine (D-Met); however, animals can only utilize L-Met. D-Met absorbed into the animal body must be converted to L-Met before it becomes active. D-Met needs to be oxidized by D-amino acid oxidase (DAO, EC: 1.4.3.3) into 2-keto-4-methylthioburyric acid, which is then transaminated by aminotransferases (tyrosine aminotransferase [EC: 2.6.1.5] and aspartate aminotransferase [EC: 2.6.1.1]) [3]. Supplemented D-Met is known to be well utilized in most animals except primates [4]. The absorption rate of D-amino acids in the small intestine is similar to that of L-amino acids [5]; however, a recent study showed that the transport system of D-Met might be more efficient than that of L-Met [6]. The bioavailability of D-Met is similar to that of L-Met when small amounts of D-Met are added to practical diets; however, it usually becomes lower with the presence of other D-amino acids in purified diets [4]. Lewis and Baker [4] summarized and reviewed the current literature, and they determined that the relative bioavailability of D-Met compared to L-Met was 76–100% in mice, 84–100% in rats, 74–100% in chickens, 50 (very young; 10–14 d of age)–100% in pigs, 14–100% in humans, and 100% in dogs. Differences between the bioavailability of L-Met and D-Met may become even greater when the supply of methionine is crucial (e.g., early stages of growth with higher growth rates).

Recently, the average temperature during summer has risen due to climate change. High ambient temperature can cause heat stress in animals, and the deleterious effects of heat stress are more prominent in broilers with higher growth rates [7]. Heat stress induces oxidative stress via reactive oxygen species (ROS) production [8]. It has been reported that methionine supplementation increased antioxidant activity (i.e., increased GSH level and higher glutathione peroxidase and catalase activity), and thus reduced ROS-induced damage in quails [9] and broiler chickens [10, 11]. Del Vesco *et al*. [12] examined the expression of selected protein deposition-related genes after methionine supplementation in acute heat-stressed broilers. They reported that methionine supplementation could reduce protein degradation induced by heat stress by increasing the expression of IGF-1 and GHR, and decreasing the expression of CTSL2 and atrogin-1 [12]. However, previous studies have not investigated whether methionine isoforms differ in their ability to mitigate oxidative stress induced by heat stress. We hypothesized that L-Met supplementation might be utilized more efficiently in acute heat-stressed broilers compared to D-Met, since the former can be used directly without oxidation and transamination.

The aim of the present study was to compare the effects of L-Met and D-Met supplementation on the expression of the transcriptomes of blood, ileum, and liver in broilers, using RNA-seq. We also investigated whether these effects changed under acute heat stress conditions.

## Materials and methods

This study was carried out in strict accordance with the recommendations in the Guide for the Care and Use of Laboratory Animals of the Korean Association For Laboratory Animals (KAFLA). The protocol was also approved by the Committee on the Ethics of Animal Experiments of the Chungnam National University (Approval Number: CNU-00779). All sampling was performed after euthanasia, and all efforts were made to minimize suffering.

### Animals, experimental design, diets, and management

A total of 240 one-day-old Ross 308 broiler chicks (47.50 ± 0.20 g) were obtained from a commercial hatchery (Joinbio, Yongin, Republic of Korea). The experiment was divided into two consecutive periods, with each period (120 birds per period) being of five weeks duration within the same facility under the same experimental protocol. Ten broilers were housed in raised wire-floor pens (0.85 m × 0.55 m × 0.35 m). In the first period, the control group was kept under typical thermo-neutral conditions, with the environmental temperature at 30 ± 1°C from day 1 to 3, gradually decreasing to 25 ± 1°C until 14 d of age, and maintained at 25 ± 1°C throughout the rest of the experimental period. An acute heat-stressed group (HS) was utilized for the second period. The environmental temperatures of the HS group were the same as that of the control group from phase 1 until 14 d of age. On the 14^th^ day, the temperature was increased to 33°C for 2 h, maintained at 33 ± 0.5°C for 3 h, and then returned back to normal temperature (25 ± 1°C). The deep-body temperature of the birds during the acute heat stress period (start, 2 h, and 5 h) was measured using a thermistor probe (FlashCheck^®^ Tip Probe Thermometer, Weber Scientific, Hamilton, NJ 08691) inserted into the bird’s rectum. Heavy panting, dispersed distribution within the cage, and significantly higher rectal temperature ensured us that the birds were sufficiently heat stressed (data were shown in previous paper [13]).

Each group of birds was randomly allocated to one of two dietary treatments: with either L-Met or D-Met supplementation. Although DL-Met is commonly used in the field, we used 100% D-Met to compare the physiological consequences of application of its two isoforms. The experimental treatments of this study were as follows: the control group supplemented with L-Met, the control group supplemented with D-Met, the heat-stressed group supplemented with L-Met, and the heat-stressed group supplemented with D-Met based on a 2 × 2 factorial arrangement. Each treatment had six replicates with 10 birds per pen. Since the environmental temperature differed by experimental period, the temperature effect was confounded with the period effect. A basal diet composed mainly of conventional feed ingredients, such as corn, barley, and soybean meal, was formulated to maximize methionine deficiency while meeting the other nutrient requirements for Ross broilers. Either crystalline L-Met or D-Met was supplemented into the basal diet to prepare the experimental diets (S1 Table). Pens had wire floor (0.85 × 0.55 × 0.35 m^3^) and were equipped with two nipple drinkers and a metal trough. The experimental diet and drinking water were provided *ad libitum* throughout the experiment. The average daily feed intake and body weight change of the birds were measured until collection at 14 days of age. There was no significant difference in feed intake and body weight gain between treatments (*P* > 0.05).

After heat treatment, all chicks were treated with the same procedure and raised up to five weeks of age in order to investigate the effects of acute heat stress on growth performance. The detailed experimental procedure can be found in a previous study [13].

### Sample collection

One chick per pen for each treatment (six chicks/treatment) was randomly selected after heat stressed chicks were exposed to high temperatures (33°C for 5 h). The chicks were euthanized via cervical dislocation to collect blood, ileum, and liver samples. Blood samples were collected in EDTA tubes from the jugular vein of each chicken. Ileum and liver samples from each chick were collected in 50 ml Falcon tubes. Blood and tissue samples were immediately transported to the laboratory for total RNA isolation.

### Total RNA extraction

Both tissues were homogenized using a mortar and pestle after being frozen in liquid nitrogen prior to RNA extraction from liver and ileum samples. Total RNA was extracted from the tissues using a RNeasy Mini Kit according to the manufacturer’s instructions (Qiagen, Hilden, Germany). The RNA from whole blood was extracted via QIAamp RNA Blood Mini kit (Qiagen, Hilden, Germany). Total RNA concentration and purity (OD 260/280 ratio) were determined using a Nanodrop 2000 spectrophotometer (Thermo Scientific, DE, USA). The quality of the RNA was also checked using an Agilent Bioanalyzer 2100 (Agilent Technologies, CA, USA). Only RNA samples with OD 260/280 between 1.8 and 2.2 and RNA Integrity Number (RIN) greater than 7 were used for RNA sequencing. The RNA samples were diluted to 50 ng/μl using RNase-free water. Two RNA samples from each treatment group were then randomly selected and pooled in equal amounts. Subsequently, a total of 36 RNA samples (triplicates per three tissues per treatment group) were obtained and stored at -80°C to minimize degradation before RNA sequencing.

### cDNA library preparation and RNA sequencing

According to the manufacturer’s manual, the cDNA libraries were constructed using TruSeq RNA Sample Prep Kit (Illumina, San Diego, CA, USA). Constructed cDNA libraries were amplified by PCR and purified using 1% formaldehyde-agarose gel. The sequencing libraries were prepared by TruSeq 3000/4000 SBS Kit v3 (Illumina, San Diego, CA, USA) and were sequenced via HiSeq4000 platform (Illumina) with 101 bp of paired ends.

#### Quality control and mapping of reads

First, the RNA-seq reads of each sample were differentiated (i.e., demultiplexed) according to their indexing adaptors, and then processed with FastQC v0.10.1 (http://www.bioinformatics.babraham.ac.uk/projects/fastqc/) to check the quality of the raw sequence reads. The reads were mapped to the chicken reference genome build, Gallus_gallus-5.0, using TopHat v2.0.4 [14], a fast splice junction mapper for RNA-seq reads. The parameters of TopHat were set to only allow a unique alignment to the reference genome. Reads with more than two mismatches were discarded, and concordant mapping for both reads in each pair was required. The alignment BAM files were further examined using RNA-SeQC v1.1.7 to obtain the mapping statistics [15].

### Counting mapped reads and identification of differentially expressed genes (DEG)

Gene-level mapped reads were counted using HTSeq-count (v.0.6.1p1) based on their alignment to the chicken reference genome build, Gallus_gallus-5.0 (ftp://ftp.ensembl.org/pub/release-86/gtf/gallus_gallus/Gallus_gallus.Gallus_gallus-5.0.86.gtf.gz) [16]. The strand parameter was set to no (-s no) because RNA-seq libraries were not constructed to be strand-specific, and the other parameters were set with default values. The count data of these mapped reads were used to identify differentially expressed genes (DEG) via R package DESeq2 (v.1.14.0) [17]. For statistical analysis on various aspects of DEG identification, Chi-square likelihood ratio tests (LRT) were performed to analyze the significance of the deviation between the full model and the reduced model.

#### All interaction

To see if there are apparent gene expression patterns differed by tissue, heat stress, and the form of supplemented methionine, a statistical analysis was conducted for the interaction between the combination of tissue and heat stress and the form of supplemented methionine. The linear model used for this analysis is as follows:

$$\text{Full}\;\text{model}:y_{ijk}=\mu +\alpha_{i}+\tau_{j}+\alpha \tau_{ij}+e_{ijk}$$

$$\text{Reduced}\;\text{model}:y_{ijk}=\mu +\alpha_{i}+\tau_{j}+e_{ijk}$$

Where: *y*_*ijk*_ is the *k*th observation in the *i*th combination of tissue and heat stress and *j*th methionine supplementation, *μ* is the overall mean, *α*_*i*_ is the fixed effect of the *i*th combination of tissue and heat stress (*i* = 1 to 6), *τ*_*j*_ is the fixed effect of the form of supplemented methionine (D-Met or L-Met), *ατ*_*ij*_ is the fixed effect of the interaction between the combination of tissue and heat stress, as well as the form of supplemented methionine, and *e*_*ijk*_ is the unexplained random error.

#### Tissue-specific effects of methionine isoform

Tissue-specific DEG between the two forms of supplemented methionine after the effect of heat stress was accounted for. The following linear model was tested for each tissue (i.e., blood, ileum, and liver):

$$\text{Full}\;\text{model}:y_{ijk}=\mu +\rho_{i}+\tau_{j}+e_{ijk}$$

$$\text{Reduced}\;\text{model}:y_{ijk}=\mu +\rho_{i}+e_{ijk}$$

Where: *y*_*ijk*_ is the *k*th observation in the *i*th heat stress treatment, and *j*th methionine supplementation, *μ* is the overall mean, *ρ*_*i*_ is the fixed effect of heat stress (control or heat stress), *τ*_*j*_ is the fixed effect of the form of supplemented methionine (D-Met or L-Met), and *e*_*ijk*_ is the unexplained random error.

#### Tissue-specific interaction between heat stress and methionine isoform

For each tissue (i.e., blood, ileum, and liver), the interaction between heat stress and the form of supplemented methionine was analyzed, and the following linear model was tested:

$$\text{Full}\;\text{model}:y_{ijk}=\mu +\rho_{i}+\tau_{j}+\rho \tau_{ij}+e_{ijk}$$

$$\text{Reduced}\;\text{model}:y_{ijk}=\mu +\rho_{i}+\tau_{j}+e_{ijk}$$

Where: *y*_*ijk*_ is the *k*th observation in the *i*th heat stress treatment, and *j*th methionine supplementation, *μ* is the overall mean, *ρ*_*i*_ is the fixed effect of heat stress (control or heat stress), *τ*_*j*_ is the fixed effect of the form of supplemented methionine (D-Met or L-Met), *ρτ*_*ij*_ is the fixed effect of the interaction between heat stress and the form of supplemented methionine, and *e*_*ijk*_ is the unexplained random error.

To assess the statistical significance of DEGs, generated *P*-values were adjusted using the Benjamini-Hochberg method (BH) to correct the false positive rate (FDR) test [18]. The DEGs were determined to be significant if the FDR was less than 0.05. An enrichment test was performed based on Gene Ontology (GO) and Kyoto Encyclopedia of Genes and Genomes (KEGG) pathway terms using DAVID (https://david.ncifcrf.gov/) to explain the biological function of DEGs. DAVID results with an Expression Analysis Systematic Explorer (EASE) score less than 0.05 were defined as significant.

### Real-time quantitative RT-PCR confirmations

We performed real-time quantitative reverse transcription PCR (real-time qRT-PCR) analysis for five genes (*YF5*, *SLC6A20*, *WDR17*, *DACH2*, and *LOC417937*) to verify the differential expression of DEGs identified by RNA-Seq. These genes were randomly selected from 15 DEGs that showed > 1- or < -1-fold-change differences between the two forms of supplemented methionine (L-Met vs. D-Met; Table 1). The raw total RNA underwent cDNA synthesis using BioFACT^™^ RT-Kit (BIOFACT, Daejeon, Korea) before the pooling process for RNA-seq, according to the manufacturer’s instructions. All primers were designed using the web-based primer design software Primer3 (version 0.4.0; http://frodo.wi.mit.edu/primer3/). The criteria of the primer design were set as follows; annealing temperature (Tm) from 58 to 62°C, a primer size of 20–25 mer, and a product size of 100–200 bp. The qPCR amplification was performed on an ABI 7500 Fast Real-Time PCR system (Applied Biosystems, CA, USA) using BioFACT^™^ 2X Real-Time PCR Master Mix (Biofact, Daejeon, Korea). The PCR cycling conditions were as follows: uracil DNA glycosylase activation at 50°C for 3 min with an initial denaturation step at 95°C for 15 min, followed by 40 denaturation cycles at 95°C for 20 s, annealing at 60°C for 30 s, extension at 72°C for 30 s, and one final extension cycle at 72°C for 5 min. Finally, a melting curve analysis was performed by slowly cooling the reaction mixture from 95°C to 65°C to detect nonspecific amplification products. The housekeeping gene *ACTB* (*β*-actin) was used as the internal control (Table 1). The 2^-ΔΔCt^ method was used to analyze relative RNA expression. Regression analysis was conducted to evaluate the concordance between predicted and observed DEG expression levels.

**Table 1:** Information on design of primers used for real-time qRT-PCR analysis.

Gene Symbol	Entrez Gene ID	Forward and reverse primer sequence (5ʹ-3ʹ)		Size (bp)
*YF5*	427746	GGGATTTTGACTGGTTCCTGAG	CTGTCTCCCATCTCCTCTTGGT	228
*SLC6A20*	420699	CTCACAGGAACCCTTCAGTACC	TGGAGACTCTTTCCCTCTTCAG	181
*WDR17*	422570	GTCTGTCCATACCTCGACCTTC	CTGTTCGATTTTCAACGGTACA	219
*DACH2*	395576	TAATTCTAACGCAGGTGGGAGT	TCATAAACGGCAGGTCTATCCT	191
*LOC417937*	417937	ATCCTCGATAACACCATTCCAG	GATACCGAAACCAAGAGACAGC	163
*ACTB*	396526	CAGGTCATCACCATTGGCAAT	GCATACAGATCCTTACGGATATCCA	149

## Results

### Sequencing the transcriptome, aligning, and mapping reads to the genome

A total of 1.87 billion of raw reads were obtained from 36 samples (four treatments × three composited replicates × three tissues) using the Illumina HiSeq 4000 sequencing platform (S3–S5 Tables). The average raw reads per sample were around 52.0 million reads (42.1‒72.1 million, S1 Fig). Approximately 97.9% of the raw reads (about 1.83 billion) passed quality control and were mapped into the chicken reference genome build 5.0 using TopHat2. Approximately 89.1% (about 1.63 billion) of the clean reads were then mapped to the reference genome; 92.4% (about 1.51 billion) were uniquely mapped, while 7.6% (about 0.12 billion) were mapped in multiple sites of the genome. Using HTseq-count, it was found that on average, 82.3% of the uniquely mapped reads were located within exon regions, while 16.8% were assigned as “no feature” meaning that the mapped reads could not be assigned to any gene model. In addition, 1.4% of the uniquely mapped reads were assigned to multiple genomic features, and were classified as “ambiguous” The summary statistics information of RNA-seq data is shown in S2 Table.

### Differential gene expression in response to the interaction among tissue, heat stress, and the form of supplemented methionine

A total of 258 genes were differentially expressed in response to the interaction among tissue, heat stress, and the form of supplemented methionine (FDR < 0.05). Hierarchical clustering based on their expression clearly showed distinct gene expression patterns among tissues and between treatments (S2 Fig). DEG expression in blood were different from those in the liver and ileum. Blood samples were first clustered by the acute heat stress treatment and then by the form of supplemented methionine. Unlike the blood samples, the liver and ileum samples were clustered together based on heat treatment. Within the same heat treatment, distinct gene expression patterns were observed between the liver and ileum.

### Differential gene expression after supplementation of isoforms of methionine (L-Met vs. D-Met)

**Fig 1:**
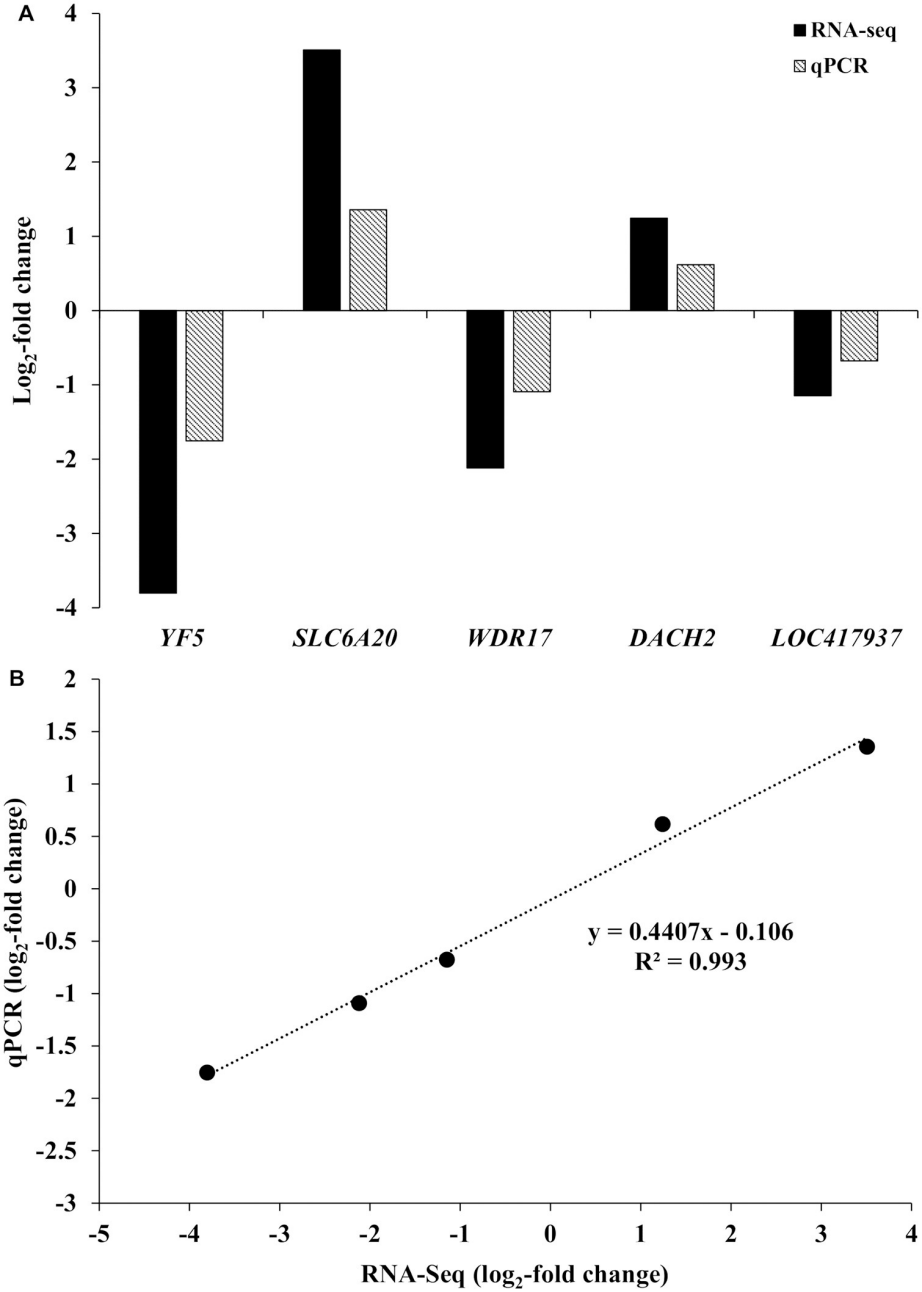
qPCR validation of differentially expressed genes (DEGs). The genes were differentially expressed in response to the interaction between heat stress and the form of supplemented methionine on whole blood, ileum, and liver. (A) X-axis represents 8 selected genes for qPCR and Y-axis represents the log_2_-fold change from RNA-seq and qPCR. (B) Regression analysis of the log_2_-fold change values between RNA-seq and qPCR.

A total of 3, 4, and 22 genes were differentially expressed for the two supplemented methionine isoforms in whole blood, ileum, and liver, respectively (FDR < 0.05). Among them, two, three, and ten genes showed greater than 1 or less than -1 log_2_ fold change difference between the two dietary treatments in whole blood, ileum, and liver, respectively (Table 2). The real-time qRT-PCR assays of the five genes (*YF5*, *SLC6A20*, *WDR17*, *DACH2*, and *LOC417937*) selected from the DEGs identified by RNA-seq showed a good agreement between the expression patterns of RNA-seq and real-time qRT-PCR results (Fig 1). The log_2_ fold-changes analyzed by the two methods were strongly and positively correlated (*r* = 0.99).

**Table 2:** Differentially expressed genes in response to two forms of supplemented methionine (L-Met vs. D-Met).

Tissue	Gene symbol	Entrez Gene ID	Gene description	Log_2_ fold change^a^	FDR
Blood	*WNK4*	777580	WNK lysine deficient protein kinase 4	1.05	<0.001
Blood	*YF5*	427746	MHC class I antigen YF5	-3.80	0.002
Ileum	*LOC107051457*	107051457	Uncharacterized LOC107051457	1.11	0.014
Ileum	*LOC107057378*	107057378	Antimicrobial peptide NK-lysin-like	-2.93	0.043
Ileum	*ATP13A5*	424902	ATPase 13A5	1.75	0.043
Liver	*LOC417937*	417937	Solute carrier family 23 member 1-like	-1.15	0.005
Liver	*GRIN3B*	429171	Glutamate ionotropic receptor NMDA type subunit 3B	-1.20	0.007
Liver	*MHCIA4*	100859807	Major histocompatibility complex, class I, A4	-1.35	0.020
Liver	*LOC107054990*	107054990	Uncharacterized LOC107054990	-1.93	0.033
Liver	*DACH2*	395576	Dachshund family transcription factor 2	1.24	0.035
Liver	*LOC107051423*	107051423	Epidermal growth factor receptor kinase substrate 8-like protein 3	-1.58	0.039
Liver	*ZNF185L*	422301	Zinc finger protein 185-like	-1.19	0.039
Liver	*SLC6A20*	420699	Solute carrier family 6 member 20	-2.58	0.049
Liver	*TLDC2*	101747236	TBC/LysM-associated domain containing 2	-1.50	0.049
Liver	*WDR17*	422570	WD repeat domain 17	2.16	0.049

^a^ The positives and negatives indicate up- or down-regulation, respectively, in the L-Met supplemented group compared to the D-Met (FDR < 0.05, fold change > 1 or < -1.

In ileum, the expression of *ATP13A5*, related to the transport of inorganic cations, and uncharacterized *LOC107051457* gene were higher in L-Met than in D-Met. On the contrary, *LOC107057378*, which performs a role in anti-microbial and anti-tumor defenses, was up-regulated in the D-Met treatment when compared with the L-Met treatment, which may indicate that anti-inflammatory responses were up-regulated in the chicks fed D-Met.

Expression of eight out of the ten DEG genes in the liver was downregulated in the L-Met treatment when compared with the D-Met treatment, while the expression of the remaining two genes was upregulated. The eight genes down-regulated in L-Met were: *LOC417937* (regulating vitamin C transport), *GRIN3B* (regulating glutamate-gated ion channels), *LOC100859807* (immune response), *LOC107054990* (uncharacterized gene), *LOC107051423* (regulating actin cytoskeleton), *ZNF185L* (regulating actin cytoskeleton and cellular differentiation and/or proliferation), *SLC6A20* (calcium-dependent uptake of amino acids), and *TLDC2* (oxidative stress resistance). *DACH2* related to myogenesis, and *WDR17* related to autosomal recessive retinitis, were the two genes that were up-regulated in the L-Met treatment when compared with the D-Met treatment.

### Differential gene expression in response to interactions between heat stress and the form of supplemented methionine (L-Met vs. D-Met)

**Fig 2:**
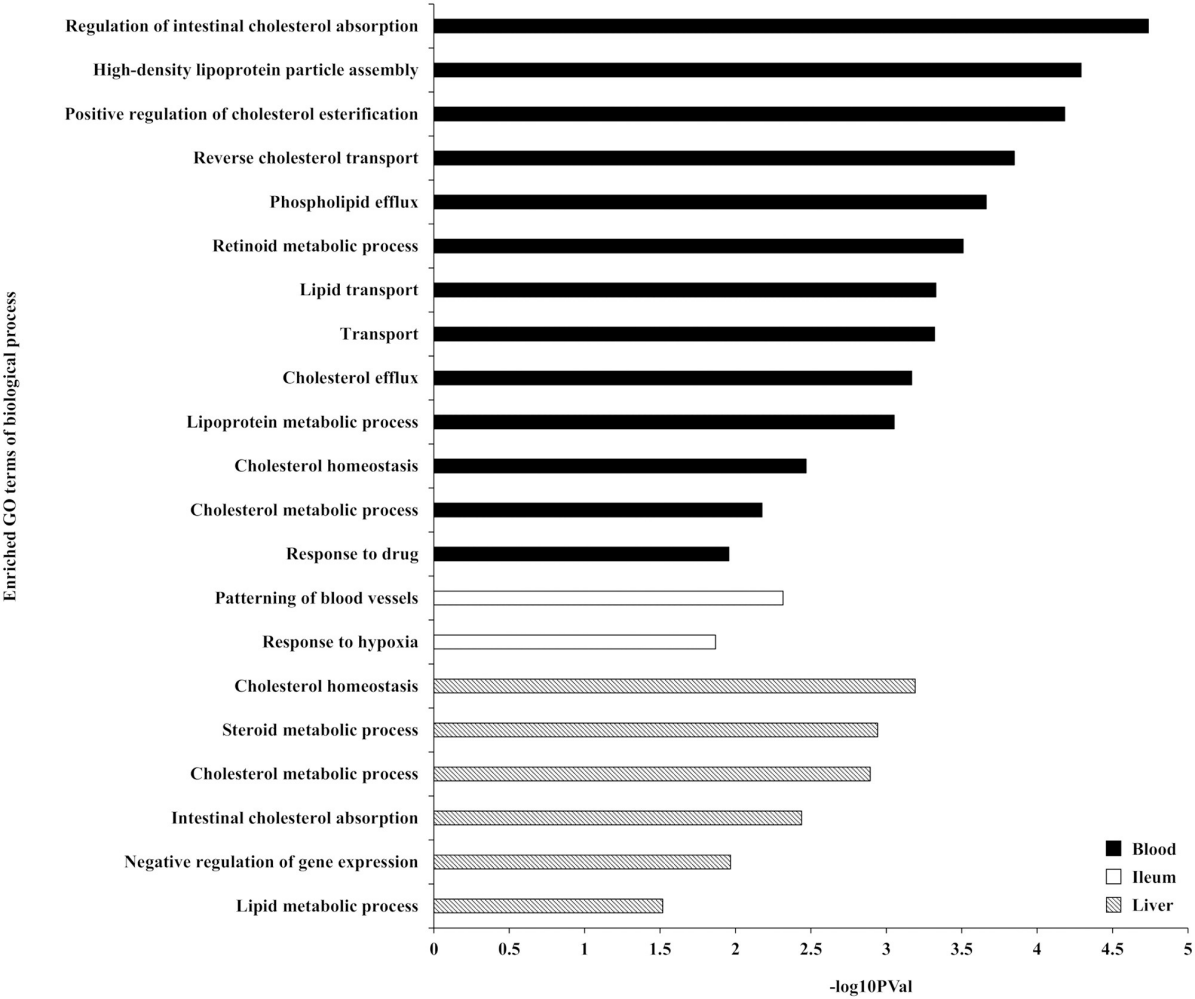
Enriched Gene Ontology (GO) terms of biological process for the differentially expressed genes (DEGs) (EASE score < 0.05). The GO enrichment analysis was performed for the DEGs in response to the interaction between heat stress and the form of supplemented methionine on whole blood, ileum, and liver.

**Fig 3:**
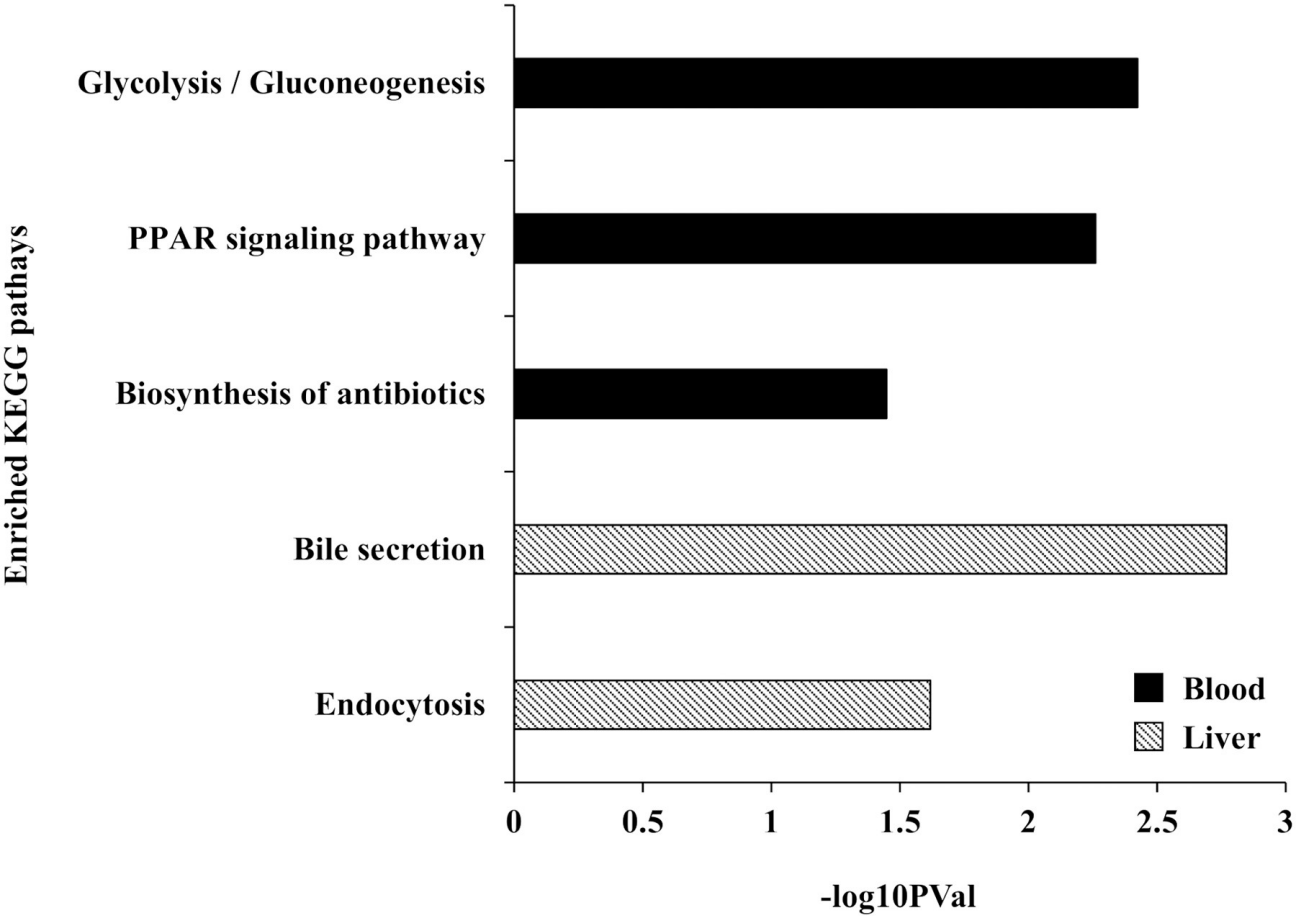
Enriched KEGG pathways for the differentially expressed genes (DEGs) (EASE score < 0.05). The KEGG enrichment analysis was performed for the DEGs in response to the interaction between heat stress and the form of supplemented methionine on whole blood and liver.

Analysis for each tissue indicated that 38, 71, and 16 genes were differentially expressed (FDR < 0.05) in response to the interaction between heat stress and the forms of supplemented methionine (L-Met vs. D-Met) in whole blood, ileum, and liver, respectively (S6–S8 Tables). Enrichment analyses for Gene ontology (GO) terms and KEGG pathways of these tissue-specific DEGs revealed that these DEGs are functionally enriched for lipid (especially cholesterol) metabolism and transport, as well as gluconeogenesis (EASE score < 0.05). The “regulation of intestinal cholesterol absorption,” “patterning of blood vessels,” and “cholesterol homeostasis” were the most prominently enriched biological processes (GO terms) in the DEGs of whole blood, ileum, and liver, respectively (Fig 2). The enrichment analysis of KEGG pathways identified three pathways in the DEGs of whole blood: “Glycolysis / Gluconeogenesis,” “PPAR signaling pathway,” and “Biosynthesis of antibiotics” (Fig 3). Two pathways for DEGs of the liver were enriched: “Bile secretion” and “Endocytosis” (Fig 3). No KEGG pathway was identified in the DEGs of the ileum.

**Fig 4:**
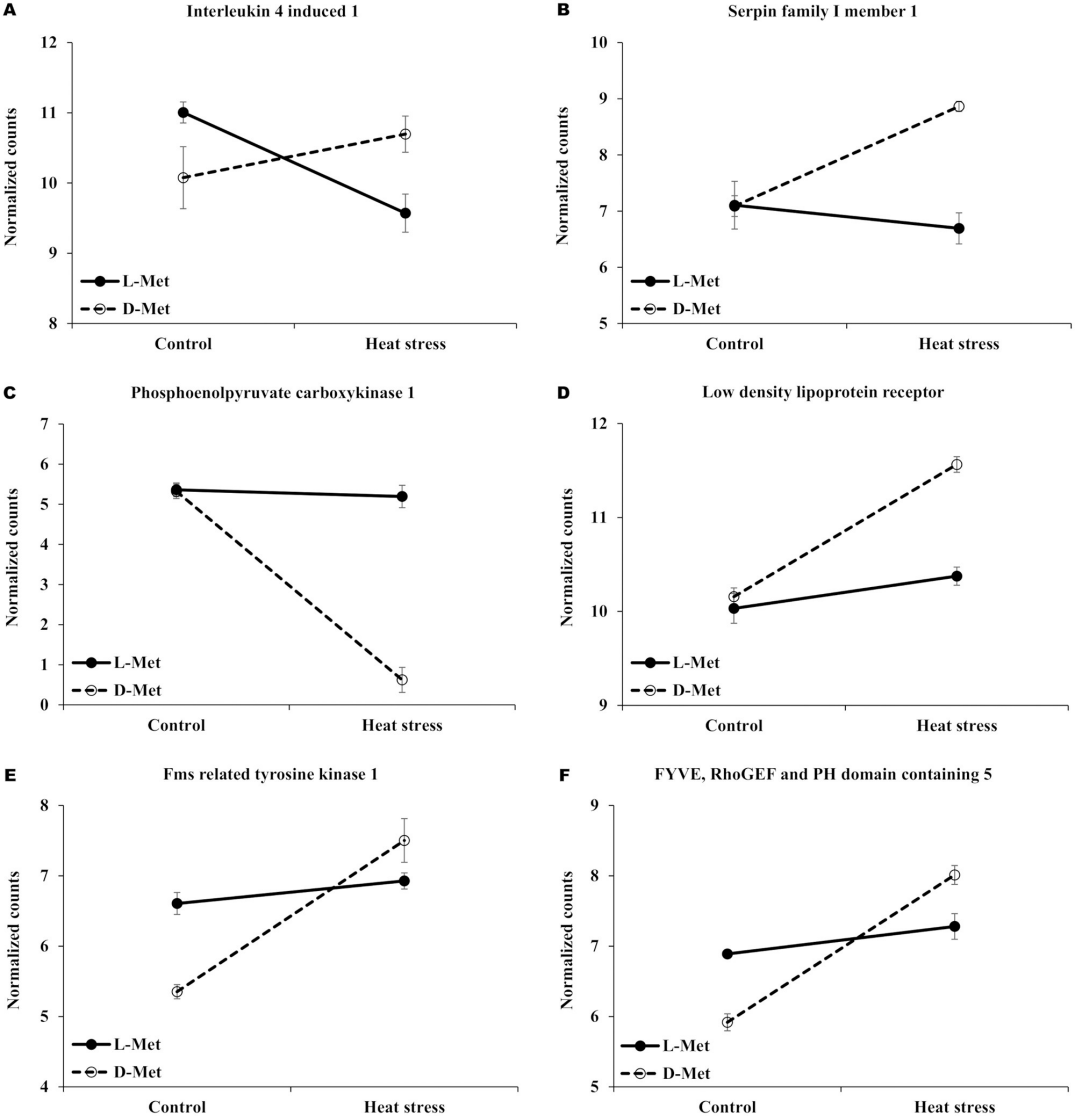
The expression patterns of the Differentially expressed genes (DEGs) (FDR < 0.05). Selected genes were differentially expressed in response to the interaction between heat stress and the form of supplemented methionine (L-Met vs. D-Met) in whole blood, ileum, and liver. L-Met, D-Met. (A) and (B) shows the selected genes related with immune response to heat stress; (C) and (D) shows selected genes related with metabolic response to heat stress; (E) and (F) shows selected genes related with intestinal response to heat stress.

The expression patterns of some of the DEGs related to immune response, metabolic response, and intestinal response to stress are presented as interaction plots in Fig 4. With these prominent DEGs, an intensive functional investigation was manually conducted based on literature. The heat stress treatment increased *IL4I1* (Interleukin 4 Induced 1) and *SERPIN1* (serpin family I member 1) in whole blood of broiler chicks fed with D-Met, which are related to immune response and neuroprotection (Fig 4A and 4B), while the expression of *PCK1* (phosphoenolpyruvate carboxykinase 1) decreased, which is a main control point for the regulation of gluconeogenesis (Fig 4C). The expression of *IL4I1* gene decreased in the L-met group, and the expression of *SERPIN1* and *PCK1* was slightly altered. The gene expression of *LDLR* (low-density lipoprotein receptor), related to the endocytosis of low-density lipoprotein, increased in the D-Met group under acute heat stress conditions, but not in the L-Met group (Fig 4D). In the ileum, the expression level of *FLT1* (fms related tyrosine kinase 1) and *FGD* (FYVE, RhoGEF and PH domain containing 5), related to angiogenesis and vasculogenesis, were relatively low in the D-Met group under normal temperature when compared with the L-Met supplemented group. The expression of these genes, however, dramatically increased in the D-Met group when exposed to a high ambient temperature (Fig 4E and 4F).

## Discussion

Methionine is an essential amino acid involved in protein synthesis as well as various physiological processes, and is routinely supplemented in animal diets. It has recently been reported that methionine supplementation can reduce protein degradation and ROS-induced damage produced due to heat stress in broilers and quails [10–12, 19]. Methionine has two isoforms (D- and L-), of which the L-form can be used immediately, but the D-form must be converted by D-amino oxidase and aminotransferase to the L-form to be active. We hypothesized that, compared with D-Met, L-Met may be used more efficiently during the early developmental stages of broilers and under acute heat stress conditions, since the former can be directly used without oxidation and transamination. Using RNA-sequencing, we examined whether the two isoforms of methionine have different effects on the expression of the transcriptome in the ileum, blood, and liver tissues, which are representative sites of absorption, transport, and metabolism of methionine in broiler chicks, respectively. In addition, we examined the interaction between the isoforms of supplemented methionine and heat stress.

There were significant phenotypic differences due to acute heat stress, which have been published previously [13]. The acute heat stress significantly reduced intake and weight gain of chickens regardless of the supplemented methionine isoform. However, the effects of acute heat stress diminished after a week. Interestingly, regardless of heat stress exposure, chickens fed L-Met had a significantly lower incidence of foot pad dermatitis, a common disease in broiler chickens, when compared with chickens fed D-Met. In the L-Met supplemented group, the numbers of birds without lesions and those with severe lesions were a 28.3%-point unit more and a 16.7%-point unit less when compared with the D-Met group.

The results of differential gene expression in response to the interaction among tissue, heat stress, and the form of supplemented methionine showed that the samples from the same tissue and the same treatment group were mostly clustered together based on the similarity of gene expression. This may indicate that the treatments could derive unique gene expression patterns, and that the tissue samples and RNA were successfully isolated.

In the blood, the expression of *WNK4* was higher, and *YF5* was lower in the L-Met supplemented group when compared with the D-Met supplemented group. The *WNK4* gene encodes WNK lysine deficient protein kinase 4 (also known as serine-threonine protein kinase), and plays an important role in the regulation of electrolyte homeostasis, cell signaling, survival, and proliferation by inhibiting the activity of the thiazide-sensitive Na-Cl cotransporter (NCC), epithelial Na+ channel (ENaC), and renal outer medullary K+ channel (ROMK). This gene increases paracellular chloride permeability [20] and causes pseudohypoaldosteronism type II, a disease characterized by hypertension with hyperkalemia [21]. The *YF5* gene is a major histocompatibility complex (MHC) class I gene conserved in many species including human, mouse, and zebrafish [22]. The MHC class I molecules are found on the surface of almost all cells, including platelets [23, 24], whose function is mainly to trigger an immune response by displaying peptide fragments of non-self-proteins in virally infected cells. Therefore, the expression of *YF5* increases with viral infections.

In this study, results from the multi-tissue transcriptomic analysis showed that immune response, lipid (especially cholesterol) metabolism and transport, and glucose metabolism were altered by the interaction between heat stress and the isoform of supplemented methionine. Compared with the D-Met supplemented group, the L-Met supplemented group appeared to have less cellular and metabolic responses to heat stress.

The expression patterns of some DEGs (*IL4I1* and *SERPINI1*) indicated that acute heat stress enhanced cellular immune responses in the D-Met supplemented group, but not in the L-Met group. When chicks were exposed to acute heat stress, the expression of *IL4I1* (Interleukin 4 Induced 1), which encodes L-amino acid oxidase and catalyzes the oxidation of L-amino acids, was significantly increased in the D-Met supplemented group, but not in the L-Met group. Zhang *et al*. [25] reported that increased expression of *IL4I1* enhanced the innate immune response and serum IgY concentration in chicken. The expression of *IL4I1* is induced by interleukin 4 (*IL-4*), which also up-regulates heat shock genes and stimulates the cellular responses under heat stress [26, 27]. Thus, the low expression of *IL4I1* in the L-Met supplemented group indicates that the cellular response to heat stress, mediated by *IL-4*, was not induced in this group. Reduced immune response to heat stress in the L-Met supplemented group was also supported by the lowered expression of *SERPINI1* (serpin family I member 1) in the L-Met supplemented group after exposure to heat stress. The *SERPINI1* gene encodes neuroserpin, which has an antioxidant defense mechanism and plays an important role in protecting neurons against oxidative stress [28]. A previous study showed that oxidative stress induced by heat stress increased the level of neuroserpin [29]. Thus, an increase in the expression of *SERPINI1* in the D-Met supplemented group implied that oxidative stress was more profound when compared with the L-Met supplemented group.

Several DEGs suggest that, under acute heat stress conditions, lipid and glucose metabolism were altered in the D-Met group, but not in the L-Met group. Oxidative stress associated with high ambient temperature is known to markedly increase the concentration of plasma glucose and cholesterol in poultry [30, 31]. It is also reported that gluconeogenesis can be enhanced in heat-stressed chicken [32, 33]. High glucose concentration in the blood reduces the expression of *PCK1*, which encodes cytosolic phosphoenolpyruvate carboxykinase, a key enzyme in *de novo* glucose synthesis [34, 35]. In the present study, the *PCK1* expression was dramatically reduced by acute heat stress in the D-Met supplemented group. This indicates changes in the glucose metabolism of the D-Met group after exposure to acute heat stress. Similarly, in consistent with our study, Ma et al. [36] reported that the expression of *PCKc* (PCK-cytosolic form) and *PCKm* (PCK-mitochondrial form) was significantly increased in the broiler group exposed to heat stress.

In addition, several key hepatic reactions related to hypercholesterolemia were up-regulated in the D-Met supplemented group when they were exposed to acute heat stress. Serum concentrations of total cholesterol and LDL-cholesterol were generally increased in broilers that undergo heat stress [37]. In the present study, although blood cholesterol was not measured, *LDLR* (low-density lipoprotein receptor) was up-regulated in the D-Met supplemented group under heat stress conditions. The *LDLR* uptake circulates LDL into the liver and regulates intracellular cholesterol levels [38]. The activation of cellular responses to reduce cholesterol concentration in the D-Met supplemented group was also indicated by the down-regulation of *GREM2* (Gremlin 2). Wu *et al*. [39] showed that the expression of *GREM2* was significantly down-regulated by a high concentration of blood cholesterol. They reported that *GREM2* inhibits adipogenesis through Wnt/β-catenin signaling. Overall, in our study the DEG expression patterns suggest that broiler chicks supplemented with D-Met had an activated cellular response to reduce circular cholesterol and glucose levels when exposed to acute heat stress conditions.

Compared with the L-Met group, intestinal angiogenic responses to heat stress appeared to be induced in the D-Met supplemented group. The expression of *FLT1* (fms related tyrosine kinase 1) and *FGD5* (FYVE, RhoGEF and PH domain containing 5) were lower in the D-Met group under normal conditions but were dramatically elevated under heat stress conditions. The *FLT1* gene is mainly expressed in vascular epithelial cells and plays an important role in angiogenesis and vasculogenesis [40]. The *FGD5* gene plays a role in the regulation of the proangiogenic action in vascular endothelial cells [41]. Heat stress has been reported to cause cellular hypoxia in intestinal tissue [42], and it is well documented that cellular hypoxia induces angiogenesis [43]. Similarly, previous studies have shown an increased expression of *FLT1* under heat stress and hypoxic conditions [44, 45].

Consequently, the current multi-tissue transcriptomic analysis showed that broiler chicks supplemented with L-Met were less stressed than D-Met when acutely exposed to high temperature. Unlike D-Met, L-Met supplementation appears to help maintain physiological homeostasis and enhance the defense system against external stresses (i.e., high environmental temperature). The discrepancy between L-Met and D-Met could be due to the higher bioavailability of L-Met and increasing doses of D-Met can potentially have similar effects. The underlying mechanism by which the L-Met supplementation reduces physiological stress produced by high temperature is still unknown. It can be speculated that the antioxidant activity of methionine, which reduces ROS-induced oxidative stress [9], was enhanced by L-Met supplementation. However, evidence to support this speculation was not found in our multi-transcriptomic analysis. In addition, the differential expressions of some genes (*IGF-1*, *GHR*, *CTSL2*, and atrogin-1) that were reported to reduce protein degradation induced by heat stress [12] were not observed in the present study. Further studies are required to understand how L-methionine supplementation can maintain physiological homeostasis against heat stress and the amount of the D- or DL-forms of methionine required to induce physiological effects similar to those induced by the L-form of methionine.

## Supporting information

S1 Fig The total number of sequence reads for each sample. (TIF)

S2 Fig The hierarchical clustering on the basis of relative expression level (in normalized counts) of differentially expressed genes (DEGs) (FDR < 0.05). The genes were differentially expressed in response to the interaction among tissue, heat stress, and the form of supplemented methionine. Abbreviations: BLD, whole blood; ILM, ileum; LVR, liver; CON, control group; HS, acute heat stressed group; L, L-Met supplementation; D, D-Met supplementation.
(TIF)

S1 Table Ingredients and chemical composition of the experimental diets. (XLSX)

S2 Table Summary statistics for sequence quality and alignment information of RNA-seq data. (XLSX)

S3 Table Statistics for sequence quality and alignment information of RNA-seq data in whole blood. (XLSX)

S4 Table Statistics for sequence quality and alignment information of RNA-seq data in ileum. (XLSX)

S5 Table Statistics for sequence quality and alignment information of RNA-seq data in liver. (XLSX)

S6 Table Normalized read counts of differentially expressed genes in whole blood in response to the interaction between heat stress and the form of methionine supplementation (L-Met vs. D-Met). (XLSX)

S7 Table Normalized read counts of differentially expressed genes in ileum in response to the interaction between heat stress and the form of methionine supplementation (L-Met vs. D-Met). (XLSX)

S8 Table Normalized read counts of differentially expressed genes in liver in response to the interaction between heat stress and the form of methionine supplementation (L-Met vs. D-Met). (XLSX)
