# Supplementary figures and images for: Multi-tissue transcriptomic analysis reveals that L-methionine supplementation maintains the physiological homeostasis of broiler chickens than D-methionine under acute heat stress
Source: PLoS One. 2021 Jan 27;16(1):e0246063. doi: 10.1371/journal.pone.0246063 (PMC7840013; doi:10.1371/journal.pone.0246063)

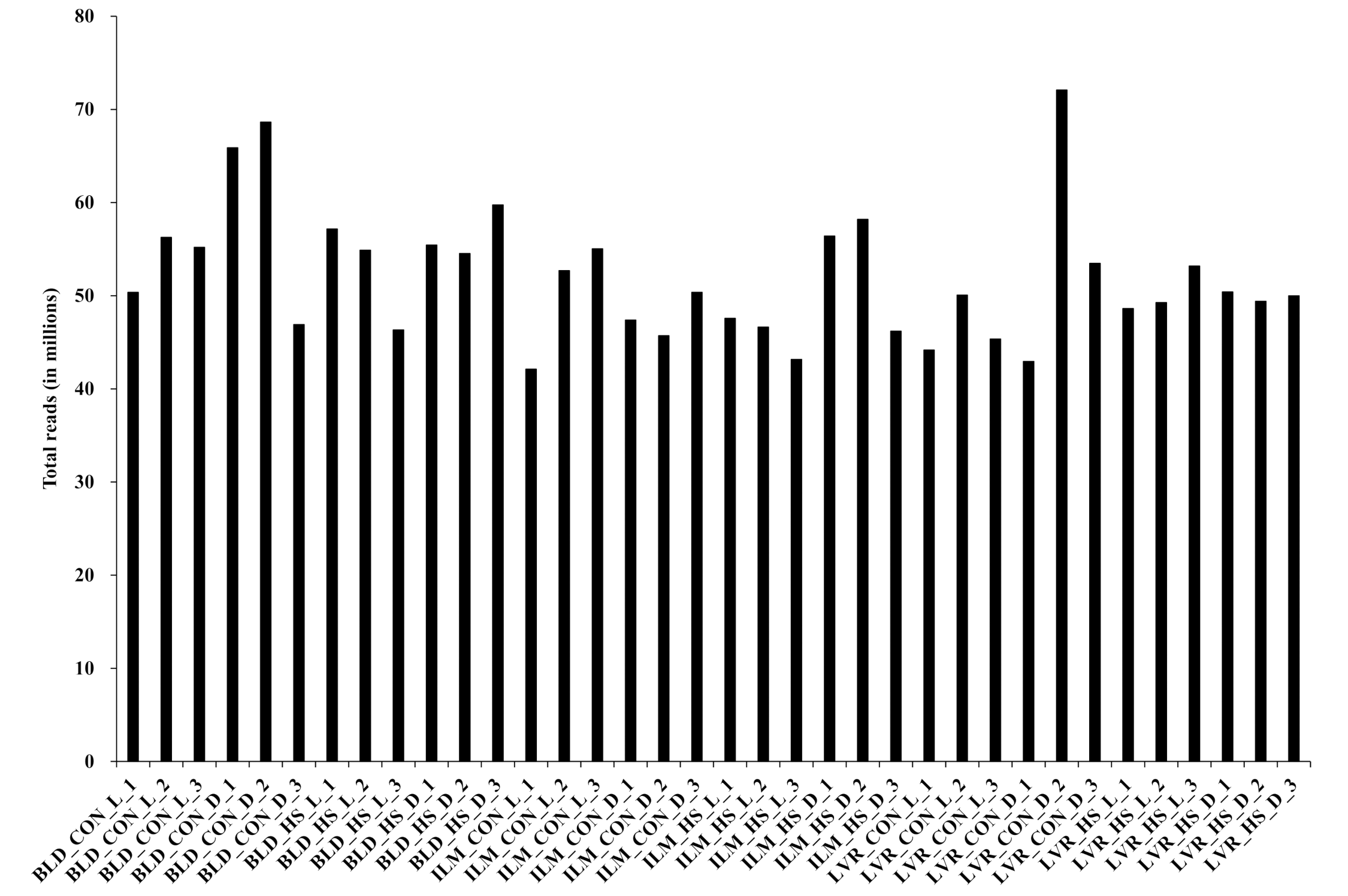

Supplement: S1 Fig — (TIF) [file pone.0246063.s001.tif]

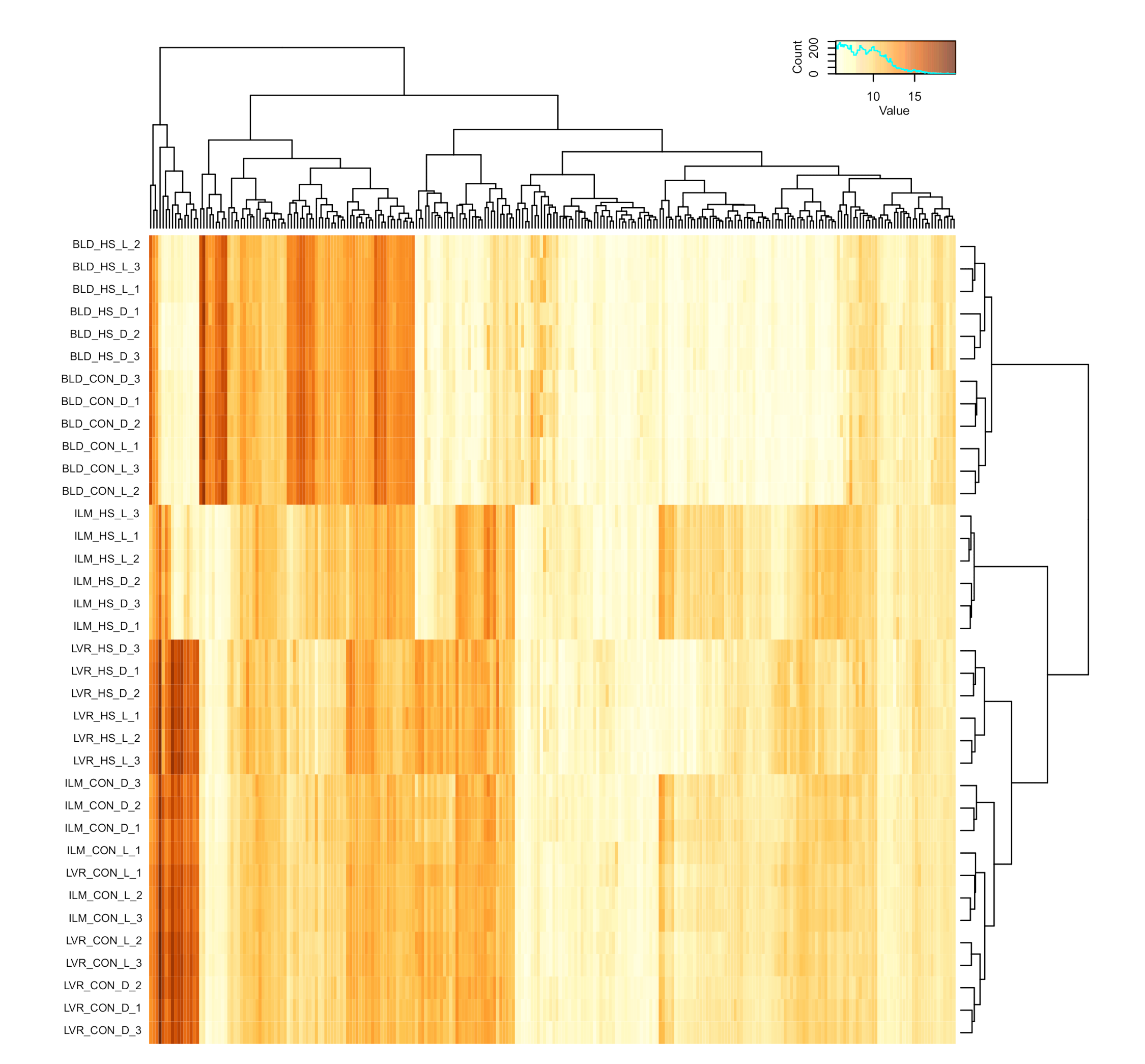

Supplement: S2 Fig — The genes were differentially expressed in response to the interaction among tissue, heat stress, and the form of supplemented methionine. Abbreviations: BLD, whole blood; ILM, ileum; LVR, liver; CON, control group; HS, acute heat stressed group; L, L-Met supplementation; D, D-Met supplementation. (TIF) [file pone.0246063.s002.tif]
